# Maintaining ethnic identity amid additive multilingualism: language attitudes and ethnolinguistic vitality among the Hmong of northern Vietnam

**DOI:** 10.3389/fpsyg.2026.1919125

**Published:** 2026-10-07

**Authors:** Jinzhi Yu, Xueyan Ma, Wei Bo

**Affiliations:** 1 School of Chinese Language and Literature, Yunnan Normal University, Kunming, China; 2 Yunnan Chinese Language and Culture College & School of International Chinese Language Education, Yunnan Normal University, Kunming, China; 3 School of Humanities, Kunming University, Kunming, China

**Keywords:** additive multilingualism, ethnic identity, ethnolinguistic vitality, heritage language maintenance, Hmong, language attitudes, minority languages, social identity theory

## Abstract

Research traditions in language attrition tend to read a minority mother tongue’s retreat from public domains as a sign of one-directional decline. This study examines that equation through the case of the Hmong in northern Vietnam, where Vietnamese dominates public institutions while Hmong contracts toward family and community, and asks whether such a retreat is necessarily subtractive. The study draws on a qualitative, reflexive thematic analysis of semi-structured interviews with six Hmong speakers across three generations, with two comparison participants offering outsider and institutional perspectives. It is framed by social identity theory, ethnolinguistic identity theory, and the perceived ethnolinguistic vitality tradition. Findings reveal a clear domain-based functional differentiation: Hmong is anchored as the first-choice language in family and ritual domains, Vietnamese dominates public-institutional domains, and the two coexist in flexible bilingual practice in intermediate domains such as markets and social media. Across generations, affective loyalty to Hmong remains stable and unmarked, even as instrumental reasoning tilts toward Vietnamese among the young. Hmong speakers’ perceived vitality forms a distinctive configuration—high cultural-status perception, low institutional-support perception, and demographic optimism—that decouples from objective institutional conditions. Contrasted with the deeper language shift documented among the Miao of Guizhou, China, this case shows that ethnolinguistic vitality is determined not by ethnic membership itself but jointly by institutional environment, demographic ecology, and community networks. The retreat of Hmong thus represents an additive multilingual configuration, marking the boundary conditions under which the attrition narrative holds and offering an empirical record of identity-driven language maintenance.

## 1 Introduction

In the mountainous regions of northern Vietnam, Vietnamese has become the default language in public institutions such as schools, hospitals, and government offices; minority mother tongues remain active in everyday life yet hold no secure place in institutional domains such as formal education (Nguyen, 2022). Among the younger generation, language choice in public domains tilts markedly toward Vietnamese, and the space for Hmong use contracts inward, toward family and community. Research traditions in language attrition and language endangerment tend to read such a retreat of the mother tongue from public domains as a sign of one-directional decline (Schmid and Köpke, 2017; Fishman, 1991). But equating a narrower range of use directly with declining vitality risks conflating two processes that ought to be kept apart: a language ceding ground in public domains, and any shift in its standing within ethnic identity and emotional life, need not move in the same direction or at the same pace.

The conceptual resources for separating these two processes have long been available within bilingualism research. Landry and Allard's (1984) distinction between additive and subtractive bilingualism allows that a dominant language may be acquired at the expense of the mother tongue or, equally, be layered upon a secure first language, and Ruiz's (1984) language-as-resource orientation reframes the mother tongue from a problem to be overcome into a resource worth maintaining. Together they recast the question from whether Hmong is retreating to whether that retreat is subtractive or additive—something that cannot be settled from the public-domain distribution of languages alone, but requires attention to how speakers themselves perceive and evaluate their language.

Two further frameworks, set out in full in Section 3, supply the terms for that inquiry. Ethnolinguistic vitality theory (Giles et al., 1977) identifies status, demography, and institutional support as the structural bases of a group’s capacity to sustain its language, and its perceived-vitality strand (Allard and Landry, 1986) holds that it is speakers’ subjective appraisal of these conditions, rather than the objective indicators themselves, that drives maintenance behavior; this study adopts the perceived-vitality orientation. Ethnolinguistic identity theory (Giles and Johnson, 1987), extending social identity theory (Tajfel and Turner, 1979), treats language as a core marker of the boundary between “us” and “them,” which opens the possibility that loyalty to a mother tongue is anchored in identity rather than in practical utility and may therefore behave differently from its range of use.

Set against the Hmong of northern Vietnam, this possibility falls squarely within two research gaps that have yet to meet. The first concerns the object of study. Research on minority languages within Vietnam has largely centered on the ethnic groups of the Central Highlands, exposing the tension between language attitudes and mother-tongue maintenance in the school domain (Nguyen and Hamid, 2016, 2018). The Hmong communities of the northern border, by contrast, have rarely been brought under systematic study of language attitudes and vitality. Existing work on the region sits more in ethnic politics and history (Rumsby, 2022), and Hmong-language research is centered elsewhere, notably on heritage-language education among diaspora communities in the United States (Her, 2024). The second concerns findings. Vitality research on the Hmong/Miao has mostly traced a “low-vitality” trajectory, most typically in the urbanizing, diasporic context of Guizhou, China, where the younger generation tends to treat the dominant language as an instrument of socioeconomic advancement (Li et al., 2024; Shen et al., 2021). Whether the border settlement, cross-border traffic, and different national language-policy environment of northern Vietnam might give rise to another trajectory has so far gone empirically unanswered.

Drawing these threads together, this study asks whether the narrowing of Hmong use in public domains should be read as evidence of one-directional language decline, or whether the expansion of Vietnamese may instead coexist with continued mother-tongue attachment and ethnolinguistic vitality. The frameworks above allow either outcome; which obtains in the northern Vietnamese Hmong communities examined here is an empirical question, addressed through three research questions: (1) How are language attitudes and perceived ethnolinguistic vitality distributed across generations and domains among the Hmong of northern Vietnam? (2) Against the backdrop of Vietnamese expansion, how do Hmong speakers position their mother tongue in relation to ethnic identity, and through what resources and identity logics is that position sustained or eroded? (3) What does the resulting configuration imply for the language-attrition/endangerment narrative?

To address these questions, the study draws on semi-structured interviews with eight participants—six of them Hmong speakers, the other two comparison participants, a commune official who married into the community (H7) and a Kinh education officer (H8)—and proceeds along a cumulative line of argument. It first sets out the division of labor between Hmong and Vietnamese under a domain-based functional differentiation; it then traces the generational shift in the markedness of language attitudes; it uses the “same ethnic group, different trajectory” contrast with the Miao of Guizhou, China to throw the particularity of this case into relief; and on that basis it draws the findings together through perceived vitality and ethnolinguistic identity theory, opening a critical dialogue with the attrition/endangerment framework.

## 2 Research background

The Hmong stand among the larger ethnic minorities of Vietnam, numbering close to 1.4 million at the 2019 national census and ranking as one of only six minority nationalities whose population passes the one-million mark (General Statistics Office of Vietnam, 2020), with their settlement concentrated overwhelmingly in the high mountains of the northern border provinces of Hà Giang, Điện Biên, Sơn La, Lào Cai, and Lai Châu. The Hmong language belongs to the Hmong-Mien (Miao-Yao) family and stands wholly apart from Vietnamese, an Austroasiatic language, so that the two share no mutual intelligibility and mark a sharp linguistic boundary between the minority and the national majority. Lào Cai, the province in which the present fieldwork was carried out, adjoins the Chinese province of Yunnan along a frontier of roughly 180 kilometres. The Hmong are its single largest ethnic minority, at over a fifth of the provincial population, a demographic weight that sustains dense everyday use of the language even as schooling, administration, and public services operate in Vietnamese. At the same time, reported literacy among Hmong aged fifteen and above sits near 54 percent, and senior-secondary attendance well below a third (General Statistics Office of Vietnam and Committee for Ethnic Minority Affairs, 2020). These figures register the limited reach of formal institutions into the community and supply the material backdrop against which participants appraise the standing of their mother tongue.

The comparison that runs through the later analysis rests on the historical and linguistic kinship between these Hmong and the Miao of Guizhou in southwest China. Miao is the official ethnonym applied in China to a category that gathers several distantly related Hmong-Mien-speaking groups, among them the Hmu, the Qo Xiong, the A-Hmao, and the Hmong proper, and the Hmong of Vietnam descend from populations of this last subgroup that moved southward out of the mountainous provinces of southwest China principally from the eighteenth century onward (Rumsby, 2022). China records roughly eleven million Miao, more than a third of whom live in Guizhou (Population Census Office of the State Council, 2022), making that province the demographic heartland of the group, so that the Guizhou communities share ancestry, core linguistic features, and a wide range of cultural practices with the Hmong across the border while inhabiting a different national polity, contending with Mandarin rather than Vietnamese as the dominant language, and following a distinct course of urbanization and internal migration. Setting the two populations alongside one another holds ethnic origin broadly constant and allows the divergence in their language situations to be read against differences of political environment, demographic ecology, and community network rather than against ethnicity itself, which is the comparative logic developed in the Discussion.

Participants form their attitudes within a specific structural setting. Language attitudes are not isolated individual psychology but are embedded in an intergroup structure of status and solidarity (Feng et al., 2023). In Vietnam, this structure takes the form of a language hierarchy with Vietnamese as the national language: minority mother tongues, though constitutionally recognized, have long been marginalized within formal institutions such as education (Nguyen and Hajek, 2022), an institutional asymmetry that resonates with broader questions of language and power (Razmjoo Moghadam and Barani, 2025). The language practices and perceptions reported in Section 5 should be read against this structural pressure.

## 3 Theoretical framework

This section follows the four theoretical threads anchored in the Introduction and explains how they are organized into a framework that empirical analysis can work with. As shown in Figure 1, the four modules connect in sequence from background to analysis: ethnolinguistic vitality theory sits at the top; it descends, through the three-dimensional Subjective Ethnolinguistic Vitality Questionnaire (SEVQ) scaffold, to dimensions that can be probed; ethnolinguistic identity theory then links these to language loyalty; and the whole is read through the lens of the language-as-resource orientation, together feeding into three sets of findings—domain-based functional differentiation, intergenerational attitude change, and the configuration of perceived vitality. The specific role of each module is set out below.

**Figure 1:**
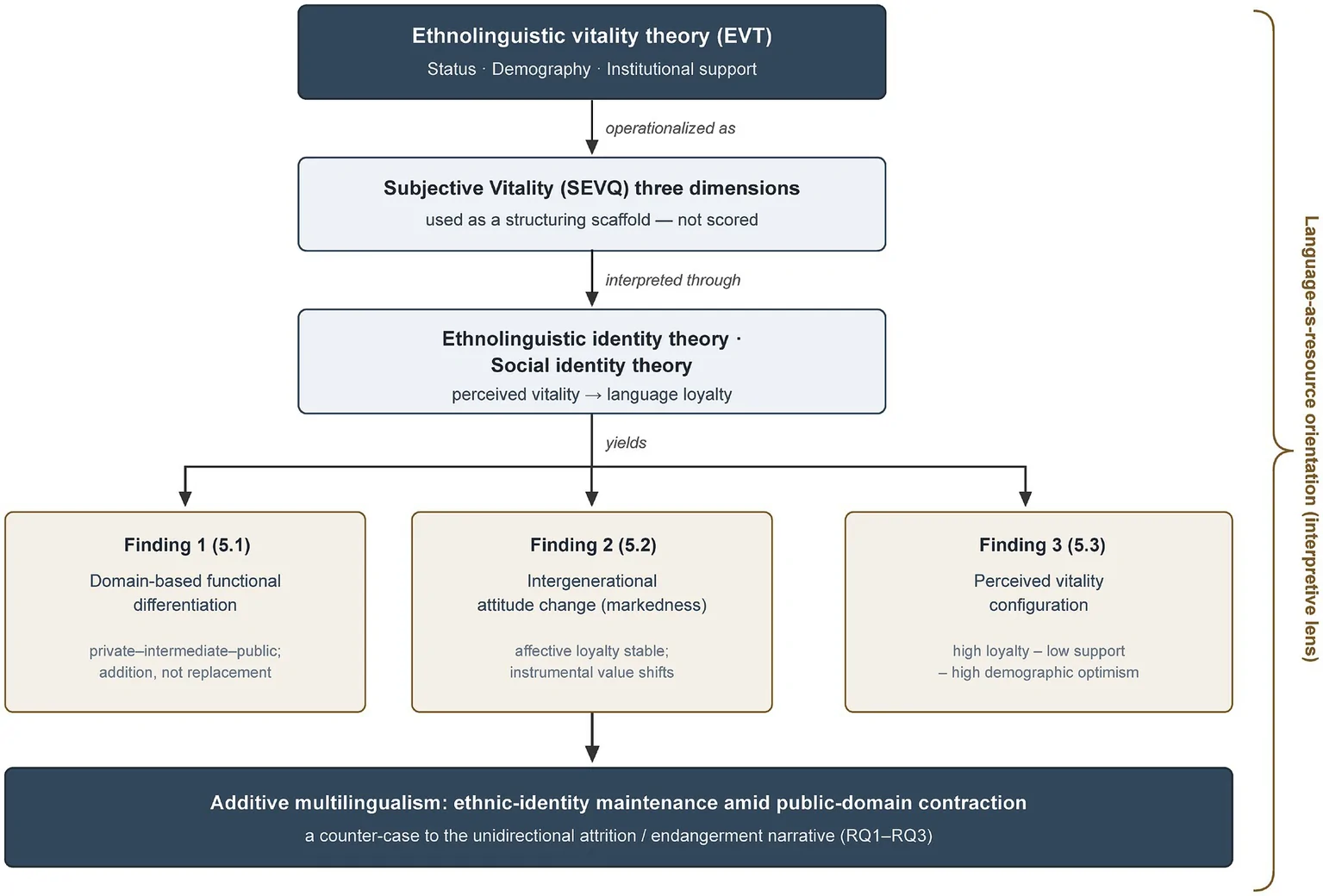
Theoretical framework anchoring the study.

### 3.1 Ethnolinguistic vitality theory and perceived vitality

Ethnolinguistic vitality theory defines the capacity of a language group to sustain its own collective linguistic behavior in intergroup settings as “vitality,” and breaks it down into three sets of structural factors: status, demography, and institutional support. In principle these can be characterized through external indicators such as census data, policy texts, and the linguistic landscape (Landry and Bourhis, 1997). What actually drives language-maintenance behavior, however, is often not these objective indicators themselves but group members’ subjective appraisal of vitality. Subjective vitality can be understood as a belief system about the present and future situation of one’s own group’s language: it both reflects reality and, by shaping members’ expectations about prospects, feeds back into their language choices (Allard and Landry, 1986). An observable gap therefore often opens up between subjective vitality and objective indicators, and the predictive power of the former over language attitudes has found empirical support (Bourhis and Sachdev, 1984). The central concern of this study is not how high or low Hmong’s objective vitality is, but how Hmong speakers read the situation of their language along the three dimensions of status, demography, and institutions; it accordingly takes a perceived-vitality orientation and does not measure objective vitality.

### 3.2 Ethnolinguistic identity theory and the social identity tradition

What allows perceived vitality to convert into durable language loyalty is the marking function language serves at intergroup boundaries. Ethnolinguistic identity theory holds that language is often used as a core marker dividing “us” from “them,” so that loyalty to the mother tongue is not merely a communicative preference but at the same time a defense of a positive group identity (Giles and Johnson, 1987). The deeper grounding of this claim lies in social identity theory: group members are motivated to maintain a positive and distinctive group identity, and achieve such positive distinctiveness through markers that foreground in-group characteristics (Tajfel and Turner, 1979). Treating language as the most salient of such markers is an extension of the social identity framework by ethnolinguistic identity theory, not a direct claim of the former; and it is along this extension that a possibility crucial to the present case opens up: Hmong’s ceding ground to Vietnamese on the instrumental level does not necessarily weaken its standing on the identity level—so long as language loyalty is anchored in ethnic identity rather than practical utility, it can hold high even as the range of use contracts. This possibility frames RQ2.

### 3.3 The language-as-resource orientation

If the first two modules explain why loyalty can be sustained, the language-as-resource orientation governs how this study reads the content of that loyalty. Ruiz (1984) distinguished three orientations toward minority languages: treating them as a problem to be overcome, as a right to be safeguarded, or as a resource worth maintaining. The resource orientation neither presupposes, as the problem orientation does, that the mother tongue is an obstacle to development, nor stops at the institutional claims of the rights orientation; instead it treats the mother tongue’s multiple functions as themselves an asset. The study adopts this orientation to provide an interpretive frame for participants’ reconstruction of the value of Hmong—speakers do not make a single choice between an “affective” and an “instrumental” pole, but reinterpret the value of Hmong along cultural, social, and affective dimensions at once. The resource orientation also lends conceptual support to the possibility of additive multilingualism: where the mother tongue is construed as an irreplaceable resource, acquiring Vietnamese may become a gain layered on top of that resource rather than a replacement for it. Scholarship since Ruiz has extended this orientation well beyond its original affirmative framing. Ruiz (2010) himself later reoriented the notion to separate the intrinsic worth a language holds for its speakers from its extrinsic value as a good exchangeable in wider markets. Subsequent commentary has folded economic capital, educational provision, and the politics of recognition into the resource frame, while cautioning that an uncritical appeal to languages as assets can slide into a neoliberal calculus that prices a tongue only by its market yield (Hult and Hornberger, 2016). This widened reading fits the Vietnamese setting, where minority bilingualism has been argued to serve social mobility and mother-tongue maintenance at once rather than to constitute a deficit (Nguyen and Hamid, 2018). It lets the value of Hmong be traced across cultural, economic, and identity registers together. The orientation adopted here therefore treats the mother tongue not as a sentimental residue set against a useful national language, but as a multidimensional asset whose cultural and affective worth is not cancelled by the instrumental gains that Vietnamese brings.

### 3.4 The three SEVQ dimensions as a structuring scaffold

The three vitality dimensions outlined in 3.1—status, demography, and institutional support—need a workable interface for empirical analysis, which this study realizes through the SEVQ. Bourhis et al.’s (1981) SEVQ operationalizes vitality along precisely these three dimensions, and the study draws on it to design its semi-structured interview guide and to organize its coding around the three, but it does not administer the instrument as a scored scale, for two reasons. Psychometrically, the validity testing of Abrams et al. (2009) shows that the SEVQ holds up as a single-dimension measure but lacks sufficient structural validity to separate three latent factors, so scoring and comparing the three dimensions separately on that basis has no robust warrant. By design, imposing a scaled administration on a small qualitative sample such as this one (six Hmong participants) would carry no statistical-inferential weight and would also cut against the study’s aim of recovering speakers’ meaning-making. Noels et al.’s (2014) review of vitality measurement tools likewise suggests that the multidimensional structure of vitality is better suited to serving as an organizing framework for analysis than as a yardstick for precise measurement. The SEVQ is thus established here as an organizing scaffold rather than a measurement instrument, with its concrete operationalization and the corresponding honesty statement given in the Methods chapter (§4.7).

## 4 Materials and methods

### 4.1 Research design

This study adopts a primarily qualitative, descriptive design, using reflexive thematic analysis to work through the interview data from the Hmong participants. The aim is to recover how Hmong speakers construct meaning around the situation of their own language, rather than to test statistical relationships among variables. No inferential statistics were performed at any stage; secondary materials were used only for descriptive background.

The interpretive stance of a reflexive thematic analysis makes the position of the researchers part of the analytic record. The research team is based at universities in Yunnan, in southwest China, and works on the documentation of Hmong dialects across the China-Vietnam borderland, a background that supplies familiarity with Hmong linguistic structure and with the broader Hmong-Miao cultural world while leaving the team external to the particular Vietnamese Hmong communities under study, positioned as cross-border academic outsiders rather than as members of the villages concerned. That external position carried both the risk of overlooking locally taken-for-granted meaning and the advantage of analytic distance. It was managed through three measures: the mediation of a local research assistant fluent in both Hmong and Vietnamese, interviews conducted in whichever of the two languages a participant preferred, and back-translation of the resulting Chinese transcripts. In this way the trilingual reality of the borderland was handled by keeping Hmong and Vietnamese as the languages of the encounter and Chinese as the language of transcription and analysis, with the assistant bridging any distance between the team’s command of Hmong and the fine grain of participants’ speech.

### 4.2 Study site and participants

Fieldwork was carried out between March and May 2024 in Lào Cai Province, Vietnam, covering three Hmong villages—Lao Chai, Ban Pho, and Ta Phin—and the town of Sa Pa. Using purposive sampling with attention to maximum variation—across generation, gender, community role, and village—we interviewed eight participants in total, in two groups, so as to capture the range of positions from which community members appraise their language. The core group comprised six Hmong speakers (H1–H6) from the villages of Lao Chai and Ban Pho, stratified by generation into G1, G2, and G3 (two participants each, one man and one woman per cohort) and spanning a range of community roles—village ritual master, traditional embroiderer, village official, homestay operator, returned migrant worker, and student. The comparison group comprised two participants who were not native Hmong speakers (H7, H8), engaged as key informants rather than as members of the analysed community; in each case the relationship was established through local introductions rather than prior acquaintance. H7, a commune civil servant in Ta Phin who had entered the community by way of a cross-border marriage from Yunnan, occupied a partial-insider position through residence and kinship yet remained an outsider by upbringing and first language; she communicated with the team in Vietnamese, with occasional recourse to the cross-border Hmong-Miao vocabulary she shared with the villagers. H8, a Kinh officer in the district education office in the town of Sa Pa, held no Hmong and was interviewed wholly in Vietnamese, contributing the vantage of the institutions that administer schooling. The analytical roles of the two groups—evidentiary for H1–H6, referential for H7–H8—are set out in 4.3. Participant details are given in Table 1.

**Table 1:** Participant profiles (*N* = 8; Hmong participants H1–H6, comparison participants H7–H8).

Group	ID	Gen.	Sex	Age range	Education	Occupation/community role	Village	Hmong/Vietnamese proficiency (self-reported)	Dur. (min)
Hmong	H1	G1	M	60–65	No formal schooling	Farmer; village ritual master	Lao Chai	Hmong fluent/basic everyday Vietnamese	68
Hmong	H2	G2	F	45–50	Lower secondary	Homestay operator	Lao Chai	Hmong fluent/fluent Vietnamese	55
Hmong	H3	G3	M	22–28	Upper secondary	Returned migrant worker	Lao Chai	Everyday-fluent, limited vocabulary/fluent Vietnamese	58
Hmong	H4	G1	F	55–60	Primary (incomplete)	Farmer; traditional embroiderer	Ban Pho	Hmong fluent/basic everyday Vietnamese	62
Hmong	H5	G2	M	35–42	Upper secondary	Village official	Ban Pho	Hmong fluent/fluent Vietnamese	70
Hmong	H6	G3	F	18–22	Upper secondary (in school)	Student	Ban Pho	Hmong fluent/fluent Vietnamese	45
Comparison	H7	–	F	30–35	College diploma	Commune civil servant (cross-border marriage, from Yunnan)	Ta Phin	Hmong limited/Vietnamese fluent	52
Comparison	H8	–	M	40–45	University	District education-office staff (Kinh)	Sa Pa town	No Hmong/Vietnamese native	60

Gen. = generation cohort (G1–G3); Dur. (min) = interview duration in minutes. H1–H6 are Hmong participants, included in the generational (G1–G3) and vitality analysis; H7 and H8 are comparison participants (see 4.2 and 4.3) and are not included in the generational stratification or thematic synthesis. Language proficiency reflects participants’ self-reported qualitative categories rather than psychometric measurement; ages are given in ranges to protect confidentiality.

### 4.3 Data sources and their boundaries

The primary data for this study are semi-structured interviews with the eight participants introduced in 4.2, and the materials are kept strictly tiered by analytical weight. As described in 4.2, six are Hmong speakers (H1–H6) and two are comparison participants (H7, H8). The six Hmong interviews form the evidentiary basis for all interpretive conclusions on language attitudes and vitality. The two comparison interviews are used only for reference and for verifying institutional facts: they corroborate the public-domain and institutional configuration reported in Section 5 but do not enter the generational stratification, thematic coding, or vitality synthesis; the asymmetry of access created by H7’s partial-insider position was contained by anonymisation and by this tiered handling. Provisions on minority languages in the Vietnamese Constitution and the Education Law, together with demographic and ethnic-education statistics, serve as traceable secondary background, describing the institutional and demographic setting in which participants are situated. On mother-tongue education specifically, UNESCO’s framework for assessing language vitality and endangerment (UNESCO, 2003) and UNICEF’s mother-tongue-in-the-classroom initiative in Lào Cai Province (UNICEF Viet Nam, n.d) jointly indicate that Hmong has long held a limited institutional position in formal education—a background read against participants’ perceived absence of institutional support. Neither the comparison interviews nor the secondary data enter into any inference of frequency or proportion.

### 4.4 Data collection

The semi-structured interview guide was built around the SEVQ dimensions of status, demography, and institutional support (Bourhis et al., 1981), and incorporated topics such as language attitudes, ethnic identity, and intergenerational transmission, formulated as open-ended guiding questions and follow-up probes that were used flexibly according to the flow of conversation rather than asked verbatim item by item. Interviews were conducted in Hmong or Vietnamese at the participant’s choosing and lasted 45–70 min; Hmong-language interviews were assisted on site by a local research assistant fluent in both Hmong and Vietnamese, and recordings were transcribed, translated into Chinese, and checked by a second person through back-translation to control for translation bias. The study was approved by the Ethics Review Committee of Yunnan Normal University (approval no. YNNU-2024-035). Given participants’ varying literacy levels and the qualitative fieldwork setting, oral informed consent was obtained from all participants before recording, as approved by the committee; the data were anonymized, and every participant is presented under a pseudonym. Beyond the interview recordings, the researchers kept field notes recording naturally occurring language practices in the settings where fieldwork took place, among them the choice between Hmong and Vietnamese at village markets and in family-run homestays, the language of exchanges at commune offices and in the presence of school-age children, and the code-switching that accompanied everyday intergenerational interaction within households. This observation was unstructured and contextual rather than governed by a formal checklist or coded as an independent data stream, since its purpose lay in situating and cross-checking the interview accounts rather than in generating counts of its own. Where an observed pattern converged with what participants reported—as in the routine switch to Vietnamese once an interaction moved into an administrative frame—the field note enters the analysis only as corroborating context. It is rendered as researcher paraphrase, never as verbatim quotation, and carries no weight in the coding frequencies. This supplementary observational layer strengthened the triangulation described below by anchoring participants’ self-reports in the observable language ecology of the villages and the town.

### 4.5 Data analysis and coding

Analysis followed the procedure of reflexive thematic analysis (Braun and Clarke, 2006, 2019): repeated reading to gain familiarity with the material, then open coding, then deriving subthemes around the three SEVQ dimensions and distilling themes from them. Two researchers coded independently and then compared line by line, discussing disagreements until consensus (consensus coding); consistent with the methodological stance of reflexive thematic analysis, the study does not aim for a quantitative intercoder-reliability coefficient but treats divergences between coders as a resource for deepening interpretation. Coding was applied only to the data from the six Hmong participants and yielded three themes and nine subthemes, whose structure and representative codes are shown in Table 2; the language-as-resource orientation is not treated as a parallel theme but as an interpretive lens running through all three themes, developed together in the Discussion (§6.2); the code frequencies in the table mark only how salient a theme is in the data, serving presentation rather than quantitative measurement. Policy and historical texts were reviewed separately along a diachronic line, serving background description only.

**Table 2:** Thematic coding framework (3 themes, 9 subthemes).

Theme	Subthemes	Representative codes (examples)
T1 Domain-based functional differentiation	Hmong anchoring in private domains; Vietnamese expansion in public domains; bilingual addition rather than replacement	Intimate family communication; monolingualization of administrative language; bilingual code-switching in markets
T2 Intergenerational attitude change	Cross-generational stability of affective loyalty; generational increase in instrumental rationality; latent fractures in transmission intent	Hmong identity as inseparable from language; Vietnamese and social mobility; gap between expectation and reality
T3 Decoupling of subjective vitality from objective reality	Cultural base of high status perception; perceived absence of institutional support; communal basis of demographic optimism	Cultural pride; governmental indifference; perceived village-wide ubiquity of Hmong

Each theme comprises three subthemes and their associated codes; the codes listed are illustrative rather than exhaustive. Codes were derived solely from the six Hmong participants’ transcripts.

### 4.6 Sample sufficiency and quality assurance

Sample sufficiency is argued here through “information power” rather than thematic saturation (Malterud et al., 2016): the aim is focused, the core sample (six Hmong participants) is highly specific, the work is guided by established theory, and the interviews—conducted in participants’ mother tongue, with concrete and detailed accounts—together mean that a smaller sample is needed (Braun and Clarke, 2021). Quality was safeguarded through triangulation (cross-referencing interviews, policy texts, and macro-level statistics), two-person consensus coding, and thick description of key contexts. The limitations of sample size and a single region are addressed further in the Discussion.

### 4.7 A key honesty statement

This study uses the three-dimensional SEVQ framework as a scaffold for organizing qualitative inquiry and coding, not as a scored scale to be administered; all judgments about vitality in the text are therefore perceived vitality as reflected in participants’ accounts, not measurement results, and no scale scores or proportions derived from them are reported. The synthesis of vitality and attitudes rests on the six Hmong participants (H1–H6) alone; H7 and H8, as comparison participants offering outsider and institutional-insider perspectives, do not count toward the vitality judgments concerning the Hmong participants. On this basis, the study’s claims to a “contribution” are confined to what is qualitative, region-specific, and perception-based.

## 5 Findings

The language practices of the Hmong participants fall along a clear gradient from private to public domains, and the overall pattern is additive rather than subtractive: Hmong holds a secure first-choice position in the family and ritual domains, Vietnamese dominates in the domains of public institutions, and the two form a flexible bilingual coexistence in intermediate domains such as markets and media. Along this gradient, language attitudes shift across generations in a discernible change of markedness, while Hmong speakers’ perception of their own language’s vitality comes systematically decoupled from the objective institutional environment they inhabit. The three sections below present these three sets of findings in turn; the synthesis of vitality and attitudes rests on the six Hmong participants (H1–H6), with the comparison participants (H7, H8) serving as the institutional-side and outsider reference only in setting out the public-domain language configuration.

### 5.1 Functional profile: domain-based functional differentiation

In the family and intimate social domains, Hmong is the first-choice language and is nearly impossible to displace, and this choice carries an identity meaning beyond that of a communicative tool. An older participant who doubles as the village ritual master (H1, G1) read the choice of language within the home directly as a measure of ethnic belonging: “At home we speak only Hmong. If my son comes back from outside and speaks Vietnamese to me, I feel he’s no longer one of us.” The irreplaceability of the ritual domain is more absolute still: even a young participant who had done urban migrant work and reported his own Hmong vocabulary eroding (H3, G3) insisted that ancestral worship must be in Hmong: “The ancestors don’t understand Vietnamese. If you call on them in Vietnamese, they won’t answer you.” Even in the youngest generation, Hmong retains priority for emotional expression—a student (H6, G3) described it this way: “When I’m angry or really happy, what comes into my head is Hmong. Vietnamese always has a layer in between; it can’t quite get that feeling across.”

The public-institutional domains present the opposite configuration, and this study’s grasp of that configuration owes precisely to the differing positions participants occupy within it. For ordinary speakers, Vietnamese dominance in the public domain registers most directly as the lived experience of bearing the cost of communication: a homestay operator (H2, G2) recalled the language gap in the medical setting—“When you see a doctor you have to speak Vietnamese; the doctor doesn’t understand Hmong. Sometimes the pain was bad and I didn’t even know how to say it in Vietnamese, so I just bore it.” Seen from inside the institutions, the same configuration is experienced by grassroots Hmong cadres as a structural dilemma: as a village official, H5 (G2) was clearly aware in the interview of Hmong’s marginalization at the policy level, describing his position as caught between “those above requiring Vietnamese and the ordinary people below who can’t understand it.” The two comparison participants (H7 and H8) further sketch, from the external and institutional sides, the Vietnamese monolingualization of the administrative and school domains—administrative business is transacted in written Vietnamese, and school instruction is conducted entirely in Vietnamese. Three observations from three different positions point to one phenomenon: Hmong lacks an institutional place in formal public domains, which therefore become the dominant space of Vietnamese.

The private and public domains do not stand in a zero-sum relation; rather, a flexible additive bilingual practice arises in intermediate domains such as markets and media. H1 described the language switching at market day as almost automatic: “When an old Hmong woman comes to buy my vegetables, I speak Hmong; when I run into a Kinh person or someone I don’t know, I switch to Vietnamese. My head just turns over by itself.” Social media has given rise to a new written-Hmong practice: H6 reported that “on Facebook I message my Hmong friends in Hmong, spelling it out with Vietnamese letters, and when chatting with everyone I use Vietnamese too—often mixing the two.” H3, away for migrant work, likewise kept up Hmong use through phone calls and gatherings of fellow villagers. The bilingual coexistence in these domains is not one language replacing another but an additive division of labor that follows the function of the domain. The private–intermediate–public functional gradient is shown in Figure 2.

**Figure 2:**
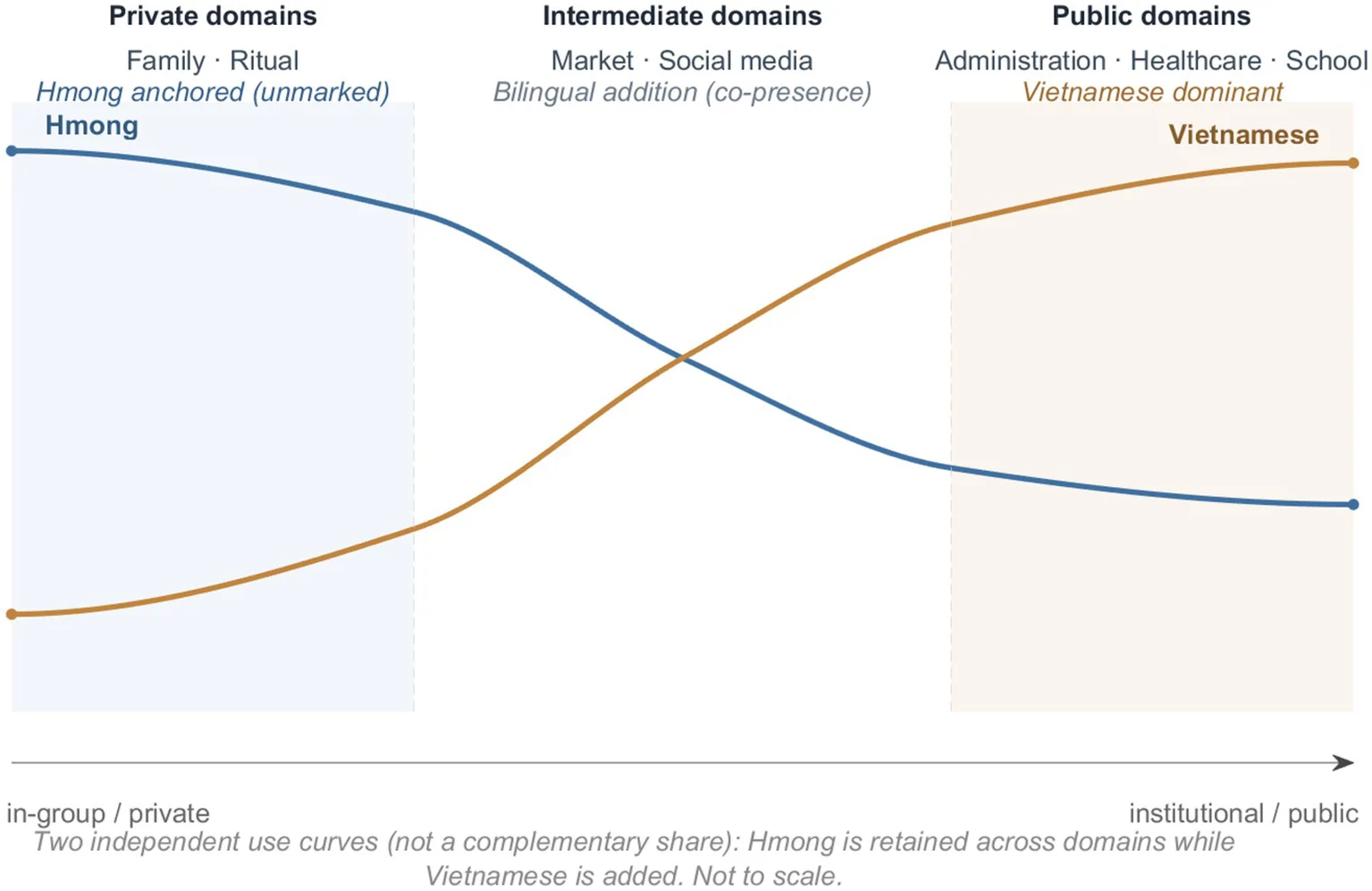
Domain-based functional differentiation between Hmong and Vietnamese.

### 5.2 Intergenerational attitudes: change in markedness

Along this domain configuration, the language attitudes of the three generations show a reorganization of markedness: within the family and the ethnic group, Hmong remains the unmarked default and it is the use of Vietnamese that carries marked significance; whereas in instrumental appraisal oriented toward social mobility, this marking relation gradually reverses across generations. Affective attachment is highly consistent across the generations and remains unmarked—using Hmong needs no reason. A middle-aged participant (H2, G2) linked transmission of the mother tongue to the ancestors recognizing their descendants: “My parents taught me Hmong, and I have to teach my children, so that our ancestors can recognize their own descendants.” The village official (H5, G2) put it more emphatically: “Hmong is the soul of us Hmong people—it’s in your bones. If you’re born Hmong, you have to speak Hmong.”

Against this stable affective loyalty, the balance of instrumental reasoning tilts clearly toward Vietnamese, and the tilt grows more open the younger the participant. H3, returned from migrant work, appraised the economic value of Hmong almost coldly: “Farming takes skill, getting a loan takes Vietnamese, and going out to work you have even less use for Hmong. What’s Hmong worth?” In domains oriented toward schooling and employment, giving Vietnamese priority is gradually turning from a “marked” act requiring explanation into a norm needing no justification—a strategic orientation that recurs across participants and is especially pronounced in the accounts of the younger generation.

It is precisely at the juncture of transmission intent that a latent fissure opens between sentiment and action. Participants almost uniformly say they want their children to keep Hmong, yet they generally concede that the environment is no longer something an individual can control. H2’s sense of powerlessness is representative: “Of course I want my children to speak Hmong, but these days they’re at school and outside all day—that environment, there’s nothing you can do about it.” Intergenerational friction sometimes surfaces directly within the household; H6 relayed her younger sister’s reaction: “When my mom tells her to speak Hmong, she even says Mom is old-fashioned and that she can’t understand what she’s saying.” The older embroidery artisan (H4, G1), facing the erosion of Hmong among her grandchildren, framed the rift as a restrained compromise: “Seeing that my grandson barely knows Hmong, of course it hurts. But I can’t blame him—times are different, and you can’t let the language hold the child back.” This rift has not yet broken transmission, but it shows that Vietnamese is gradually acquiring unmarked default status in the peer interactions of the younger generation, while the maintenance of Hmong rests increasingly on conscious effort at the level of identity rather than on the natural support of any domain.

### 5.3 Subjective vitality: a high-loyalty perceptual configuration

Placing these attitudes back within the ethnolinguistic vitality framework, the Hmong participants’ perception of their own language’s vitality forms a configuration decoupled from their objective circumstances. On the status dimension, participants affirmed the cultural value of Hmong with near-unanimous strength, and this affirmation is rooted explicitly in cultural self-assurance rather than socioeconomic prestige. H6, drawing on history and art, asked rhetorically: “We Hmong have thousands of years of history, our language is so rich, all those patterns and those songs—who dares say our culture isn’t high?” She went on to reject the notion that languages rank above one another: “Some people say Hmong is country talk, backward talk. I don’t agree. Language is language—how can there be high and low, noble and base?” For H1, speaking Hmong connects directly to ethnic dignity: “The Hmong have already taken plenty of scorn out there; if we don’t even speak our own language, we can hold our heads up even less.”

On the institutional-support dimension, however, participants’ perceptions are uniformly low. The elderly H1 summed up this absence as government inaction: “The government says it respects minority languages, but where is the actual action? I’ve lived more than sixty years and never seen the government do a thing for Hmong.” The scarcity of Hmong in the media came up repeatedly as well; H2 noted that “there has never been a Hmong channel or a proper Hmong program on television.”

Perception on the demographic dimension, by contrast, turns optimistic, grounded in the density of the Hmong population at the village level and the high everyday visibility of Hmong. H3, taking village size as his evidence, asserted that Hmong will not disappear: “Our village is four hundred-some households, all Hmong. Step outside and it’s all Hmong. You think Hmong could disappear? No way.” The village official H5, in a slightly hyperbolic register, stressed the binding of Hmong’s survival to the group’s survival: “How could Hmong ever disappear—unless every Hmong person were dead?” Taken together, the three dimensions constitute a high-loyalty, low-support, high-optimism perceptual configuration. What calls for caution is that this demographic optimism, grounded in everyday village visibility, may mask the intergenerational transmission rift exposed in 5.2—perceptual optimism and behavioral, latent loss being two sides of the very same decoupling.

## 6 Discussion

The findings of this study point to a judgment that runs counter to the attrition narrative: in the northern Vietnamese Hmong communities examined here, the retreat of Hmong from public domains has not been accompanied by a decline along the identity dimension but instead coexists with a highly stable ethnolinguistic loyalty. The four sections below place this judgment, in turn, back into dialogue with theory and the existing literature: first establishing the “additive rather than subtractive” argument through the China–Vietnam Hmong contrast (6.1), then explaining how identity can be sustained as the range of use contracts (6.2), next accounting for how the Vietnamese context shapes this configuration (6.3), and finally delimiting the study’s limitations and boundaries of applicability (6.4).

### 6.1 Additive rather than subtractive: a counterexample to the attrition narrative

What matters about the domain-based functional differentiation set out in the Findings is not which domains Hmong has ceded but the manner of the ceding: the anchoring of Hmong in the family and ritual domains has not been eroded by the Vietnamese-ization of the public domain but is, on the contrary, sustained through the flexible additive use of both languages in intermediate domains. This is a relatively stable configuration—Hmong anchored in the private domain, both languages sharing the intermediate domains, and Vietnamese dominant in the public domain—hereafter the additive steady state. Such a form is hard to grasp through an attrition framework of one language replacing another. Language-attrition research largely attends to the systematic loss of mother-tongue competence under pressure from a dominant language (Schmid and Köpke, 2017), whereas the “retreat” of Hmong in this case is a functional redistribution across domains rather than a decline in competence or loyalty—closer to an additive than a subtractive bilingual configuration (Landry and Allard, 1984). Distinguishing “decline” from “redistribution” also aligns with recent rethinking of language revitalization: revitalization is not a simple reversal of past losses but a search for a more resilient path to survival under existing conditions (Wiltshire et al., 2024).

The market vendor who moves between Hmong and Vietnamese according to the customer before him, and the young speaker who writes Hmong in Vietnamese orthography and interleaves the two on social media, are drawing on one repertoire rather than switching between two bounded codes. These practices are best understood as translanguaging, which treats a bilingual’s languages as one integrated repertoire rather than two sealed systems (García and Li, 2014). Reading them in this way rather than as mere code-switching clarifies why the expansion of Vietnamese need not erode Hmong: the added language widens the repertoire on which speakers draw instead of displacing the resource already in place. This is the additive rather than subtractive logic at the centre of this account.

Setting this form against the Miao of Guizhou, China brings into view two trajectories into which the same ethnic group has diverged under different political and social environments, as shown in Table 3. Existing research on the Guizhou Miao presents a deeper language shift: the dominance of Miao in the family domain contracts, the younger generation treats Mandarin as an instrument of socioeconomic advancement, and a loosening of language from identity emerges amid urbanizing diaspora (Li et al., 2024; Shen et al., 2021). The Hmong of northern Vietnam, by contrast, sustain both the security of Hmong in the private domain and a tight binding of ethnic identity, under conditions of border settlement, cross-border traffic, and a different national language-policy environment. Notably, the education system plays a similar role at both sites—school monolingualization is the main structural driver of language shift in each—which indicates that the divergence of the two trajectories springs not from the presence or absence of educational pressure but from differences in other conditions: community demographic ecology, cross-border networks, and identity binding.

**Table 3:** Comparison of ethnolinguistic vitality trajectories: Hmong in northern Vietnam versus Miao in Guizhou, China.

Dimension	Hmong in northern Vietnam (this study)	Miao in Guizhou, China (existing literature)	Contrast
Domain use pattern	Hmong anchored in private domains, Vietnamese dominant in public domains, bilingual addition in intermediate domains	Contraction even in private domains; Mandarin dominant in market and workplace; digital domains trending monolingual	Marked difference
Intergenerational transmission	Latent fractures but no rupture; Hmong still primary in the family domain	Deeper rupture; markedly lower Hmong fluency among the younger generation	Marked difference
Identity–language relationship	Language tightly bound to identity; “one who does not speak Hmong is not Hmong” consistent across generations	Loosening of the language–identity bond; cultural customs and descent partly substitute for language	Marked difference
Subjective vitality perception	High cultural-status perception, low institutional-support perception, demographic optimism	Generally low across dimensions; more pessimistic perception of demographic trends	Partial difference
Role of the education system	Vietnamese monolingualization in schools, the main structural driver of language shift	Mandarin monolingualization in schools; boarding systems further compress family Hmong time	Similar pattern
Community language-attitude tenor	Pragmatic but not pessimistic; bilingualism positively valued among the young	Pragmatic but more pessimistic; bilingual attitudes lean toward replacement	Marked difference

The Vietnam column derives from the qualitative synthesis of six Hmong participants in the present study; the Guizhou column summarizes existing literature (Li et al., 2024; Shen et al., 2021). “Contrast” denotes a qualitative judgment and involves no quantitative comparison. The role of the education system is a similar pattern across both sites—a shared structural constraint rather than the source of trajectory divergence.

This “divergence” is not isolated within the wider landscape of vitality research. Finland’s Swedish-speaking minority, where institutional support and demographic base are relatively ample, can sustain both ethnolinguistic identity and perceived vitality at a relatively high level (Lindell et al., 2023); against this, the Hmong of northern Vietnam sustaining high loyalty under markedly weak institutional support throws into relief just how far identity and community ecology can substitute for the function of institutional support. The same resilience is seen in other heritage-language communities: sustained investment at the level of family and cultural practice can keep a mother tongue vital under unfavorable external conditions (Bilgory-Fazakas and Armon-Lotem, 2025). Together these contrasts indicate that the level of Hmong vitality is determined not by ethnic membership itself but jointly by the institutional environment, demographic configuration, and community ecology in which it is embedded.

This contrast gives the “counterexample” its concrete content: the Hmong of northern Vietnam do not prove the attrition framework wrong but mark out the conditions under which it holds—when the mother tongue is still construed, along the identity dimension, as an irreplaceable resource, and the community’s demographic ecology can still support everyday use, a retreat from the public domain does not necessarily slide into one-directional decline. Reading every narrowing of mother-tongue use as a portent of decline would miss the additive steady state as one possible stable form.

### 6.2 Why identity can be sustained as the range of use contracts

If Hmong has clearly given way on the instrumental level, how does its ethnic loyalty remain stable across generations? The clue offered by the Findings is that this loyalty is anchored in identity rather than practical utility. Ethnolinguistic identity theory suggests that when a language serves as a core marker of intergroup boundaries, holding to it is in itself a defense of a positive group identity (Giles and Johnson, 1987). The cross-generationally consistent formulation in this study—“one who does not speak Hmong is not Hmong”—is a direct expression of this mechanism. The link between identity and attitude also finds empirical support: the strength of ethnic identity can significantly shape an individual’s language attitudes toward the mother tongue (Shabtaev et al., 2024), providing corroboration for “identity-driven loyalty” beyond the present case.

As shown in §3.3 and Figure 1, the language-as-resource orientation is an interpretive lens unifying the foregoing findings rather than a parallel theme. This identity logic and the language-as-resource orientation reinforce each other. Participants’ construction of the value of Hmong exceeds the affective–instrumental binary—Hmong is understood at once as a carrier of cultural memory, as social capital for in-group trust and cross-border connection, and as an affective bond of intimacy and belonging. Understanding the mother tongue as a multidimensional resource means that acquiring Vietnamese constitutes not a replacement for Hmong but a gain layered on existing resources (Ruiz, 1984). In an environment of limited institutional support and low measurable vitality, affective attachment and cultural practice at the family and community levels may still sustain the mother tongue’s continuation, as corroborated in other heritage-language contexts: research on family language policy in Pakistan shows the protective effect of family dynamics and cultural practice on transmission (Fatima and Nadeem, 2025), while the responsibility for maintenance does not stop at the family—grassroots action at the community level can constitute a compensatory mechanism in the absence of institutional support (Inan and Harris, 2025). Grassroots practices in this case, such as elders teaching children to sing Hmong songs, accord precisely with this direction.

The robustness of the identity–language binding does not mean it bears no tension. Research on overseas Hmong communities shows that when younger generations negotiate dual cultural belonging in mainstream society, the intergenerational loss of linguistic competence can decouple from the persistence of identity—a person can identify strongly as Hmong yet no longer use Hmong fluently (McKenzie et al., 2024). Research on intergenerational transmission likewise suggests that childhood family-language experience significantly influences one’s language-policy orientation in adulthood, producing considerable variation in transmission intent across generations (Pagé and Noels, 2024). These two threads corroborate the latent rift in Findings 5.2: although Hmong loyalty in this case is stable across generations, transmission from G2 to G3 already shows early signs that identity and competence may separate. This makes intelligible the internal consistency of the three-part configuration noted in the Findings: the perceived absence of institutional support has not dissolved language loyalty because the roots of that loyalty lie not in institutions but in identity and community ecology—though these roots may not, on their own, withstand the long-term structural pressure of the educational and economic domains.

### 6.3 Continuity and a filled gap in the Vietnamese context

The findings of this study continue an established line in research on minority languages in Vietnam while filling one of its gaps. Research on language choice among the minority groups of Vietnam’s Central Highlands has shown that the division of labor among languages across domains often follows a logic of power and solidarity, with the dominant language becoming the unmarked choice in formal domains while the mother tongue sustains solidarity within the group (Nguyen and Hamid, 2021). The reorganization of markedness observed here among the Hmong of the northern border—Vietnamese becoming the unmarked default in the peer interactions of the younger generation while Hmong retreats into a marked in-group choice—is consistent with this logic, indicating that the mechanism is to some degree transferable across ethnic groups and regions.

Widening the view to a broader range of minority multilingual cases, the generality of this mechanism and the particularity of the present case come into view together. Research on the language ideologies of 1.5-generation Korean immigrants in New Zealand shows that a contraction in the range of mother-tongue use can run in parallel with the persistence of identity construction (Park, 2022), consistent in direction with this case’s pattern of “narrowing range yet undiminished loyalty.” In China’s southwest, adjacent to northern Vietnam, research on Zhuang trilingualism in Guangxi reveals the tension between education and ethnic-language agency (Wu and Silver, 2023), while research on the language policies of multilingual families in the southwest documents families’ efforts and difficulties in maintaining the mother tongue under the pressure of the dominant language (Lam et al., 2025)—the latter echoing the family-language-policy situation of the Guizhou Miao and further showing that the additive steady state of this case is not the regional norm but a product of specific conditions. Even within a linguistic landscape absolutely dominated by Chinese, the visibility of minority languages still forms part of their perceived vitality (He and Chen, 2024), which suggests that linguistic visibility in public space carries non-negligible significance for the maintenance of Hmong.

The difference from the Central Highlands case is equally telling. The border settlement and cross-border traffic of the northern Vietnamese Hmong furnish Hmong with community demographic support and cross-border use scenarios that the Central Highlands groups may not possess, which partly explains why this case displays stronger language loyalty than earlier Vietnamese research. The institutional environment, meanwhile, forms a shared constraint: minority mother tongues, though constitutionally recognized, lack substantive support in education and public services (Nguyen and Hajek, 2022), and the Vietnamese monolingualization of schools is, in this case as in the Central Highlands case, the main driver of language shift. Hmong’s current steady state thus rests more on community ecology than on policy support.

### 6.4 Limitations and boundaries of applicability

The conclusions of this study must be understood within the boundaries of its design. The core synthesis rests on qualitative interviews with six Hmong participants across two Hmong villages in Lào Cai Province (Lao Chai and Ban Pho), with one comparison participant interviewed in each of Ta Phin and the town of Sa Pa to provide external and institutional-side reference; the region and scale of the sample mean that its purpose lies not in statistical generalizability but in exposing a form of language vitality that has lacked empirical record until now. On the criterion of information power, this sample suffices to support thick description of meaning-making in a specific community (Malterud et al., 2016), but whether the additive steady state portrayed here applies to other Hmong settlement areas in Vietnam still requires further research to test.

Vitality judgments rest on participants’ perception rather than objective measurement—both the orientation of this study and its limit: perceived vitality reveals how speakers understand the situation of their own language but cannot substitute for an independent assessment of objective vitality indicators, and the decoupling between the two itself awaits tracking through a mixed design in future work. Inferences about generation call for particular caution: this study compares the discourse of three generations at a single point in time, an apparent-time design, so the generational differences presented are strictly cross-sectional differences in the current attitudes of different generations, not a direct observation of one group’s change over time; whether the latent loss from G2 to G3 will accumulate over time into a transmission rupture lies beyond what this design can determine and must await longitudinal tracking. These boundaries both delimit the study’s claims and sketch the directions for subsequent work.

## 7 Conclusion

Taking the Hmong community of Lào Cai Province in northern Vietnam as a case, this study examined language attitudes and perceived ethnolinguistic vitality against the backdrop of Vietnamese expansion. It shows that the retreat of Hmong from public domains does not constitute one-directional decline but a functional redistribution across domains—Hmong anchored in the private domain, Vietnamese dominant in the public domain, both languages sharing the intermediate domains. Under this configuration, Hmong speakers’ affective loyalty to the mother tongue and their ethnic identity remain stable across generations, and multilingual competence is construed as a resource layered on the mother tongue rather than a replacement for it; the “retreat” of Hmong thus belongs to an additive rather than a subtractive multilingual configuration.

Setting this configuration against the Miao of Guizhou, China brings into view two vitality trajectories into which the same ethnic group has diverged under different environments, and from this follows a judgment of theoretical significance: the level of ethnolinguistic vitality is determined not by ethnic membership itself but jointly by the institutional environment, demographic ecology, and community networks in which it is embedded. The “high cultural-status perception, low institutional-support perception, demographic optimism” configuration presented in this case portrays a form in which language loyalty stays high even where institutional support is absent, offering a clear instance of the claim, within ethnolinguistic vitality theory, that subjective and objective vitality may decouple: when the mother tongue is still seen, along the identity dimension, as an irreplaceable resource, and community ecology can still support everyday use, a retreat from the public domain need not slide into decline.

Theoretically, this study uses a borderland minority case to mark out the boundaries of applicability of the attrition and endangerment narratives, and provides an empirical record of additive multilingualism as a form of language maintenance. Practically, it suggests that for Vietnam’s mother-tongue education policy to take effect, it might build on the high affective loyalty and additive orientation already present in Hmong communities, gradually creating institutional space for Hmong in the educational and media domains so that community resilience gains support at the policy level. Given the constraints of sample size and region, a perception-based orientation, and a cross-sectional design, these judgments still require testing across a wider range of Hmong settlement areas and through longitudinal and mixed methods, so as to clarify how far identity-driven language maintenance can withstand long-term structural pressure.

## Data Availability

The raw data supporting the conclusions of this article will be made available by the authors, without undue reservation.
